# Voluntary exercise does not increase gastrointestinal motility but increases spatial memory, intestinal eNOS, Akt levels, and *Bifidobacteria* abundance in the microbiome

**DOI:** 10.3389/fphys.2023.1173636

**Published:** 2023-08-16

**Authors:** Peter Bakonyi, Attila Kolonics, Dora Aczel, Lei Zhou, Soroosh Mozaffaritabar, Kinga Molnár, Lajos László, Balazs Kutasi, Kumpei Tanisawa, Jonguk Park, Yaodong Gu, Ricardo A. Pinho, Zsolt Radak

**Affiliations:** ^1^ Research Institute of Sport Science, Hungarian University of Sport Science, Budapest, Hungary; ^2^ Department of Anatomy, Cell and Developmental Biology, Eötvös Loránd University of Sciences, Budapest, Hungary; ^3^ Faculty of Information Technology and Bionics, Pázmány Péter Catholic University, Budapest, Hungary; ^4^ Faculty of Sport Sciences, Waseda University, Tokorozawa, Japan; ^5^ Artificial Intelligence Center for Health and Biomedical Research, Osaka, Japan; ^6^ Faculty of Sports Science, Ningbo University, Ningbo, China; ^7^ Laboratory of Exercise Biochemistry in Health, Graduate Program in Health Sciences, School of Medicine, Pontifícia Universidade Católica Do Paraná, Curitiba, Brazil

**Keywords:** exercise, intestine, microbiome, motility, caveolae

## Abstract

The interaction between the gut and brain is a great puzzle since it is mediated by very complex mechanisms. Therefore, the possible interactions of the brain–exercise–intestine–microbiome axis were investigated in a control (C, N = 6) and voluntarily exercised (VE, N = 8) middle-aged rats. The endurance capacity was assessed by VO_2_max on the treadmill, spatial memory by the Morris maze test, gastrointestinal motility by EMG, the microbiome by 16S RNA gene amplicon sequencing, caveolae by electron microscopy, and biochemical assays were used to measure protein levels and production of reactive oxygen species (ROS). Eight weeks of voluntary running increased VO_2_max, and spatial memory was assessed by the Morris maze test but did not significantly change the motility of the gastrointestinal tract or production of ROS in the intestine. The protein kinase B (Akt) and endothelial nitric oxide synthase (eNOS) protein levels significantly increased in the intestine, while peroxisome proliferator–activated receptor gamma coactivator 1 alpha (PGC-1α), mitochondrial transcription factor A (TFAM), nuclear respiratory factor 1 (NFR1), SIRT1, SIRT3, nicotinamide phosphoribosyl transferase (NAMPT), and nuclear factor κB (NF-κB) did not change. On the other hand, voluntary exercise increased the number of caveolae in the smooth muscles of the intestine and relative abundance of *Bifidobacteria* in the microbiome, which correlated with the Akt levels in the intestine. Voluntary exercise has systemic effects and the relationship between intestinal Akt and the microbiome of the gastrointestinal tract could be an important adaptive response.

## Introduction

The interaction between the gut and brain is a great puzzle since it is mediated by very complex mechanisms. One of the important factors in this interaction is the microbiome (Gareau et al., 2011). The microbiome is very plastic: it is easily modified by nutrition (Tarr et al., 2015; Hernandez-Calderon et al., 2022), but it is also known that exercise and aging can affect the bacterial community of the gut as well (Alesch et al., 1988). The gut microbiome plays an important role in digestion, the regulation of the intestinal and systemic immune system, and the production of small molecules and short-chain fatty acids (SCFA) that can directly interact with other organs (Clark et al., 2022). There is increasing evidence that the gut microbiome is associated with neurodegenerative diseases (Bell et al., 2019), such as dementia and Alzheimer’s disease (AD) in humans (Bell et al., 2019) and in rodents (Zhang et al., 2017). The changes in the microbiome are linked to defects in synaptogenesis and cognitive impairment, such as in AD (Mitew et al., 2013).

The health of the gastrointestinal tract is mostly dependent on the smooth muscles for motility and any damage to these may have a devastating effect on the body. Besides nutrition, physical exercise is a natural method that can alter the gut microbiome and activity of the gastrointestinal tract (Abraham et al., 2019; Gubert et al., 2020; Mahizir et al., 2020). Exercise could have a direct impact on gastroenteric activity. Indeed, it has been reported that exercising mice at 0.5 mph running speed have significantly faster gastric emptying rates after 15 min of exercise than non-exercised mice; however, at slower running speed, there was no difference between exercising and control mice (Grunewald and Tucker, 1985). It has been reported that exhaustive exercise on C57BL/6 mice resulted in oxidative stress in the small intestine and alteration to the internal mitochondrial membrane that was verified by ultra-micrography analysis (Rosa et al., 2009). Indeed, exhaustive exercise damaged the ileal mucosa layer and impaired contractility (Rosa et al., 2008). On the other hand, moderate aerobic exercise training showed a protective effect against tissue oxidative stress (de Lira et al., 2008). It is suggested that mild exercise increases the diversity of microbiome (Dziewiecka et al., 2022) and changes the abundance of “good” bacterial flora, which could directly benefit brain function (Yin et al., 2022). The extensive neuroprotective effects of exercising have been extensively reviewed by Radak et al. (2016) and Quan et al. (2020).

When a Parkinson’s model was created by 1-methyl-4-phenyl-1,2,3,6-tetrahydropyridine (MPTP), it appears that exercise training may save dopaminergic neurons from MPTP damage and promote recovery from ileal pathology (Wang et al., 2023). It is clear that the gut microbiome has a direct interaction with the intestine but this relationship, especially during exercise, is vaguely understood. Therefore, in order to better understand the causative relationship of the brain–exercise–intestine–microbiome axis, we assessed brain function, intestine motility, intestinal biochemistry, and fecal microbiome analysis of voluntarily exercised (VE) rats.

## Methods

A total of 14 Sprague-Dawley middle aged (11 months; weight, 591.58 ± 60.9 g) male rats (Charles-River Laboratories, Budapest, Hungary) were divided into a control (C, N = 6) and voluntarily exercised group (VE, N = 8). The VE group had constant access to the running wheel, and the running distance was daily recorded. Before placement of the VE group into running wheel–containing cages, the motility of their intestine and maximal oxygen uptake (VO_2_max) were measured. After the experimental period, 6 weeks of wheel running, VO_2_max test, and Morris maze tests were done. Following these tests, the animals were anesthetized intraperitoneally with ketamine (Richter, concentration: 100 mg/ml)/xylazine (Produlab Pharma, concentration: 20 mg/ml) cocktail at a dose of 0.1 ml/10 g body weight and transcardially perfused with ice-cold heparinized saline. After opening the abdomen, ∼5 mm colon rings were quickly removed from the descending colon at ∼20 mm below the ileocecal junction in the proximal region as the transmission electron microscopy (TEM) sample (N = 5 per group). For intact mitochondria and total protein extract, ∼30 mm colon sections were collected.

### Motility measurements

We measured the myoelectric activity of the stomach, ileum, and cecum using transcutaneous electrical nerve recording surface electrodes to record smooth muscle electromyography (SMEMG) as described earlier (Pribek et al., 2021). The electrodes (Electrode PE Foam Solidgel, Bio Lead-Lok B Sp. Zo.o, Józefów, Poland) were fixed onto the surface of the skin with adhesive plaster (Leukoplast 5 cm, BSN Medical GmbH, Hamburg, Germany) without surgery. Ten20 conductive EEG paste (Bio-Medical Instruments, United States) was used on the skin surface to ensure proper conductivity of the electrodes. The standard electrode pairs (two electrodes) were fixed to the right and left sides of the abdominal wall. The SMEMG measurements were done before and after 6 weeks of voluntary exercise of the VE group and controls and were recorded at the same time under stress-free conditions and carried out between 9.00 and 11.00 a.m. at room temperature (24°C). The examined electrophysiological parameters were recorded for 30 min and analyzed by an online computer and amplifier system by Advanced ISOSYS Data Acquisition System (MDE GmbH, Walldorf, Germany). Electromyographic (EMG) signals were amplified by using a custom-made amplifier designed by MDE Ltd., Budapest, Hungary. In order to reduce the artifacts, we used a double-filter system. All analog signals were pre-filtered with a first-order Bessel-type low-pass filter and converted to digital signals at a sample rate of 2 Hz with a slope of 80 dB/decade. The pre-filtered myoelectric signals were then filtered further by Bessel-type bandpass filters with a frequency of 0–30 cycles per minute (cpm) with a slope of 140 dB/decade. Each filter was a digital IIR filter. The recorded signals were analyzed by fast Fourier transformation (FFT). The frequency of the electric activity was characterized in cpm, and the magnitude of the activity was described as power spectrum density (PsD). Regarding the interpretation of the physiological parameters, if the values were >1 standard deviation, they were considered outliers and excluded from the analysis as described earlier (Pribek et al., 2021).

### VO_2_max measurements

The VO_2_max measurement was conducted in the same way as had been described by Marton et al. (2015). All animals were introduced to running on the motor-driven treadmill (Columbus Instruments, Columbus, Ohio) for 5 days for 10 min per day. For each introduction session, the treadmill incline was set at 5% and the speed was gradually increased from 8 m/min to 23 m/min. The training for the exercise group lasted for 6 weeks. VO_2_max was measured for each animal, using three criteria: i) no change in VO_2_ when the speed was increased, ii) the rats no longer kept their positions on the treadmill, and iii) the respiratory quotient was (RQ = VCO_2_/VO_2_) >1.

### Morris maze test

Spatial memory of rats was evaluated by the Morris water maze test on 5 consecutive days (four trials per day) as had been reported by Koltai et al. (2011). The hidden platform was placed in the center of the circular pool, below the water level. The water temperature was set to 22°C–23°C throughout the study. Animals were placed into the Morris water maze’ pool at one of four possible startingpoints (north, south, west, or east) and 60 s were given to each animal to find the platform. The order of the starting points varied through the study.

### Western blots

The middle part of the large intestine was homogenized by using the ULTRA-TURRAX homogenizer (IKA, Staufen im Breisgau, Germany) with 10 vol of lysis buffer. The samples were electrophoresed on 6%–15% polyacrylamide (SDS-PAGE) gels. The samples were between 3 and 6 µg. The proteins in the samples were transferred into PVDF membranes. Then, the membranes were blocked with BSA (0.5%–5%) or milk (5%) for 2 h at 4°C. After blocking, the membranes were incubated with primary antibody at 4°C overnight; antibody list: eNOS 1:5000 (ab76198), SIRT1 1:3000 (ab110304), PGC1α 1:3000 (NBP1-04676), MFN1 1:1000 (sc-50330, H-65), NRF1 1:1000 (D9K6P, 46743), Akt1 1:1000 (sc-5298), NF-κB 1:3000 (ab16502, p65), NAMPT 1:3000 (ab45890), CS 1:10000 (ab96600), and SIRT3 1:1000 (10099-1-AP). The next day, the membranes were washed thrice with Tris-buffered saline–Tween-20 (TBST) at room temperature and incubated with HRP-conjugated secondary antibody for 2 h at 4°C. After this, the membranes were washed again with TBST thrice at room temperature. Then, the membranes were incubated with a chemiluminescent substrate and protein bands were visualized on X-ray films. The bands were quantified by using the ImageJ software. The relative density was calculated with reference to the housekeeping protein used by us, which was tubulin.

### Mitochondrial, cytosolic, and nuclear fraction preparation

Fractionation was performed according to Frezza et al. (2007) with minor modifications. Every step was performed at 4°C. Briefly, the fresh, fat, and connective tissue-free colon tissue was immersed in ice-cold PBS supplemented with 10 mM EDTA and minced into small pieces. The samples were digested by 0.05% trypsin for 30 min with gentle shaking and then centrifuged at 1000 *g* for 5 min. The pellet was resuspended in a 10-fold buffer volume of IB_m_1 (50 mM Tris-HCl, 50 mM KCl, 10 mM EDTA, 0.2% BSA, and 0.067 M sucrose; pH 7.4) and homogenized gently with 3–4 strokes. The homogenate was centrifuged at 600 *g* for 10 min. A part of the nucleus that included the pellet and cytosolic supernatant was reserved for Western blot analysis. The supernatant was centrifuged at 8000 *g* for 10 min that resulted in the mitochondrial pellet. The centrifugation step was repeated after IB_m_1 buffer homogenization to gain high-quality intact mitochondria. The mitochondrial pellet was suspended in the least volume of possible IB_m_2 buffer (10 mM Tris-HCl, 3 mM Tris-EGTA, and 0.25 M sucrose; pH 7.4). The protein concentration was measured using the Bradford assay (Kruger, 1994).

### ROS production

Mitochondria (0.3 mg/ml) were incubated in experimental buffer (10 mM Tris/HCl, 5 mM MgCl_2_, 2 mM KH_2_PO_4_, 20 mM EGTA/Tris, and 250 mM sucrose; pH 7.4) supplemented with 1 μM Amplex Red (excitation: 560 nm; emission: 584 nm) and horseradish peroxidase (10 IU) to assess ROS production by monitoring H_2_O_2_-induced fluorescence according to Votyakova and Reynolds (2001) with minor modifications. After measuring basal ROS production, 10 mM succinate (Suc) and/or 1 μM rotenone (Rot) were sequentially added. With succinate as the substrate, the ROS production is augmented by that related to the reverse electron flux at the level of complex 1, which can be estimated from its inhibition by rotenone. Under these latter conditions, the addition of rotenone has two potential effects at the level of complex I: increased ROS production linked to the forward flux and decrease that is linked to the reverse electron flux (Batandier et al., 2006). Calibration of H_2_O_2_ production was obtained by the addition of a known amount of H_2_O_2_. Fluorimetric assays were performed at 30°C with Fluoroskan Ascent FL fluorimeter on 96-well plates, and each point was measured in triplicate.

### Transmission electron microscopy

The samples for TEM were immersed and fixed in modified Carnovsky fixative (3.2% PFA, 0.2% glutaraldehyde, 1% sucrose, 40 mM CaCl_2_ in 0.1 M cacodylate buffer) for 24 h at 4°C. Following that, all samples were cut into two equal parts in the midsagittal plane before post-fixation in 5% glutaraldehyde/0.1 M cacodylate buffer for 12 h at 4°C. Dehydration was performed by graded ethanol series saturated with propylene oxide. Tissue integrity and orientation were checked on semi-thin sections, which were then embedded in Spurr low-viscosity epoxy resin. Tissue integrity and orientation were checked on 0.8–1 µm semi-thin (Section 2) miniblocks (1 mm^2^) that were cut from every block: “E” miniblock contained the whole mucosa (epithelium, lamina propria, and muscularis mucosa), while “M” miniblock contained the submucosa, muscularis propria, and serosa layers. “E” miniblocks (2/animal, 10/animal group) were used for morphometric examination of epithelial cells, while “M” miniblocks were used for quantification of the caveolae number in the innermost smooth muscle cells (SMCs) of the circular muscle layer (CML). Six ultrathin sections with 50–60 nm thickness per miniblock were cut using the Reichert OMU-3 Ultramicrotome and collected on Formvar (Agar Scientific, Essex, UK) coated with copper slot grids. Uranyl acetate and Reynolds’s lead citrate were used for counterstaining. A JEOL JEM 1011 transmission electron microscope equipped with a Morada 11-megapixel camera (Olympus) was used for morphometry. For the analysis of caveolae number, 2-2 neighboring innermost SMCs were selected from different sections (four SMCs/animal with two distinct localizations). Micrographs were taken at the same enlargement (×30,000) that covered the full length of the selected SMCs. The length of the plasmalemma and number of caveolae were determined by using the iTEM software measuring option.

### Morphological measurement

Morphological measurements of the mitochondria and vesicula were obtained using the APEER Online Machine Learning Platform by ZEISS (https://www.apeer.com) and Fiji (Schindelin et al., 2012). Distinct mitochondria and vesicles were traced by the defined deep learning method on electron microscopy images of the colon of adult mice (five animals, three to four independent pictures at a magnification of ×20,000) based on previous training using a defined morphological setup. Deep learning by APEER differentiated among mitochondrial classes based on the repertoire of observed morphology—Group A: normal, dense matrix with parallel narrow cristae; Group B: disordered, still dense matrix but inordinate cristae with lipid droplets inside the matrix; and Group C: swollen, extensive, or complete loss of cristae and matrix density, significant loss of internal structure. Questionable mitochondria and/or vesicula were re-evaluated. The results were exported to Fuji and the following parameters were generated: relative mitochondrial and vesicular area, mitochondrial size; perimeter; aspect ratio (AR, length-to-width ratio) as [(major axis)/(minor axis)]; form factor (FF, reflecting the complexity aspect of mitochondria) as [(perimeter2)/(4π × surface area)]. The computed values were imported into Prism 6 (GraphPad Software) for further data analysis. The statistical significance was evaluated based on a 95% confidence interval (C.I.) of the mean.

### DNA extraction and 16S rRNA gene amplicon sequencing

Both intestinal samples in addition to a freshly collected fecal pellet were rapidly frozen in liquid nitrogen immediately following removal. For long-term storage, the samples were kept at −80°C. Total DNA was isolated from each sample using the DNeasy PowerSoil Kit according to the manufacturer’s protocol (cat. no. 12888-100; Qiagen GmbH, Hilden, Germany). V3–V4 regions of the 16S rRNA gene were amplified by PCR using the following primers: 16S amplicon PCR forward primer = 5′-TCGTCGGCAGCGT CAGATGTGTATAAGAGACAGCCTACGGGNGGCWGCAG, and 16S Amplicon PCR Reverse Primer = 5′-GTCTCGTGGGCTCGG AGATGTGTATAAGAGACAGGACTAC HVGGGTATCTAATCC. The DNA library for Illumina MiSeq sequencing was prepared using the Nextera XT DNA Library Preparation Kit (Illumina Inc., CA, United States) according to the manufacturer’s protocol. The DNA library was sequenced using the Illumina MiSeq 2 × 300 bp platform with the MiSeq v3 Reagent Kit (Illumina Inc., CA, United States) according to the manufacturer’s instructions. Sequence reads were rarefied to 10,000 reads per sample prior to further analyses.

## Statistics

The group differences investigated by two-way ANOVA group means were compared by Tukey’s HSD. If data did not follow the normal distribution assessed by Shapiro–Wilk test, the Kruskal–Wallis test was applied instead.

### Bioinformatics analysis of microbiome

All bioinformatics analyses from quality trimming of reads to diversity analysis were automatically performed using Snaq (Mohsen et al., 2022), a snakemake pipeline for 16S microbiome data analysis with QIIME 2 (Bolyen et al., 2019). In this pipeline, the DADA2 algorithm (Callahan et al., 2016) was used to infer amplicon sequence variants (ASVs), which were taxonomically classified using the SILVA 128 reference database (Quast et al., 2013) from the phylum to genus level. As a measure of species diversity within samples, the Shannon diversity index was calculated based on the ASVs.

## Results

**FIGURE 1:**
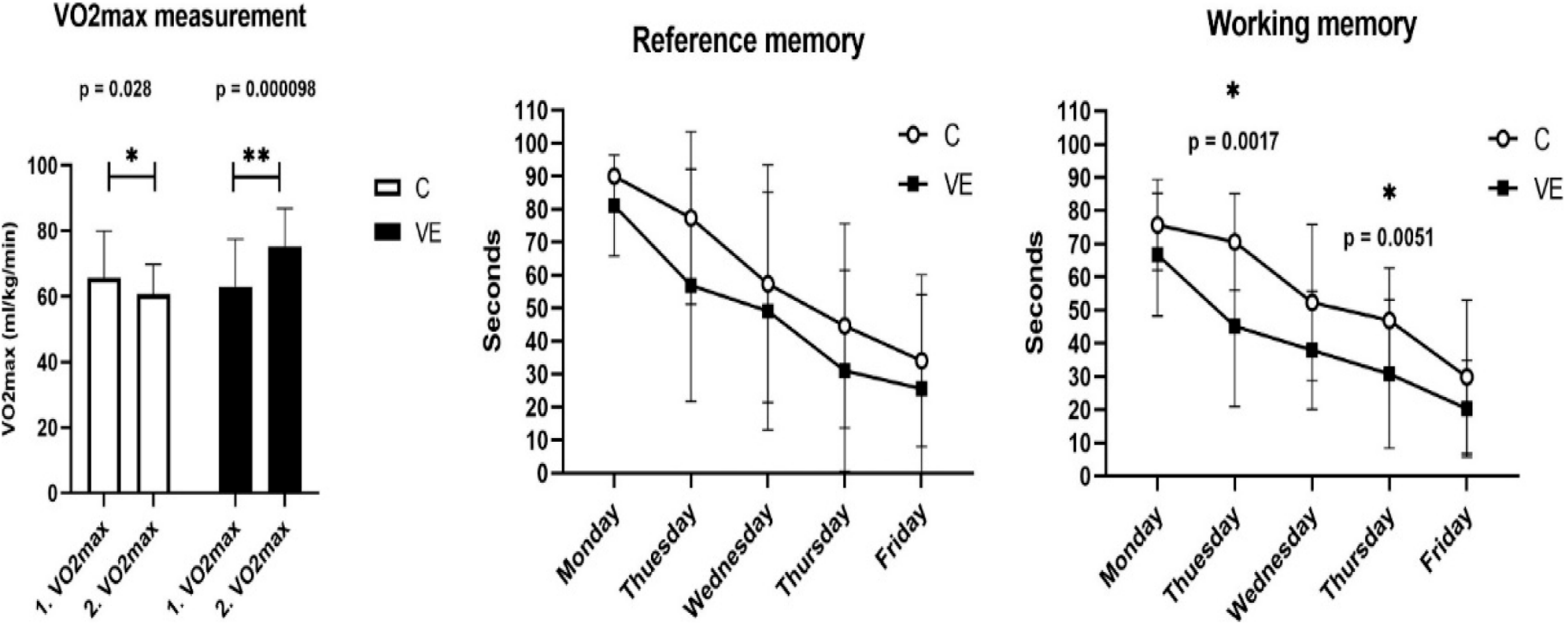
Maximal oxygen uptake, reference, and working memory of voluntarily exercised and control animals.

The VO_2_max significantly increased in the VE group when compared to the control group, while the lactate level significantly decreased immediately after the test (Figure 1A). Animals in the VE group found the hidden islands in the Morris maze test in a shorter period of time than did the control animals; however, a significant difference was only observed on the second and fourth day (Figures 1B, C).

Voluntary exercise increased the relative maximal oxygen uptake, and it significantly improved the working memory, assessed by using the Morris maze test on the second and fourth day of the 5-day trial. Working memory was calculated as the average of four trials spent in water each day, while reference memory was calculated as the average of the first trials each day. The results are given as mean ± SD of the control (C, N = 6) and voluntarily exercised (VE, N = 8) rats. Statistical significance was assessed using the two-way ANOVA.

**FIGURE 2:**
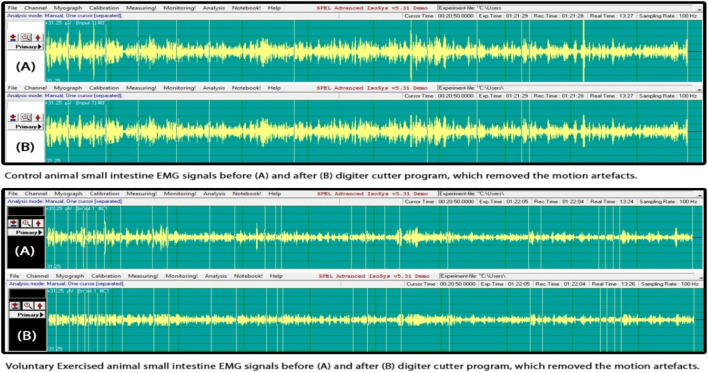
Smooth muscle electromyography (SMEMG) signals of control and exercised rats.

The results of the SMEMG measurements were similar in both the VE and control groups. However, in all measured regions (the small intestine, large intestine, and gastric part), SMEMG signals were smaller in the VE group, mainly due to the significant deviation of signals. A significant relationship was not found (Figure 2).

There was no significant difference found in the electric signal activity of the small intestine of the control and exercised rats. The evaluation of the signals was done following the method by Pribek et al. (2021). The representative signals of the control (N = 6) and VE (N = 8) animals are shown in Figure 2.

**FIGURE 3:**
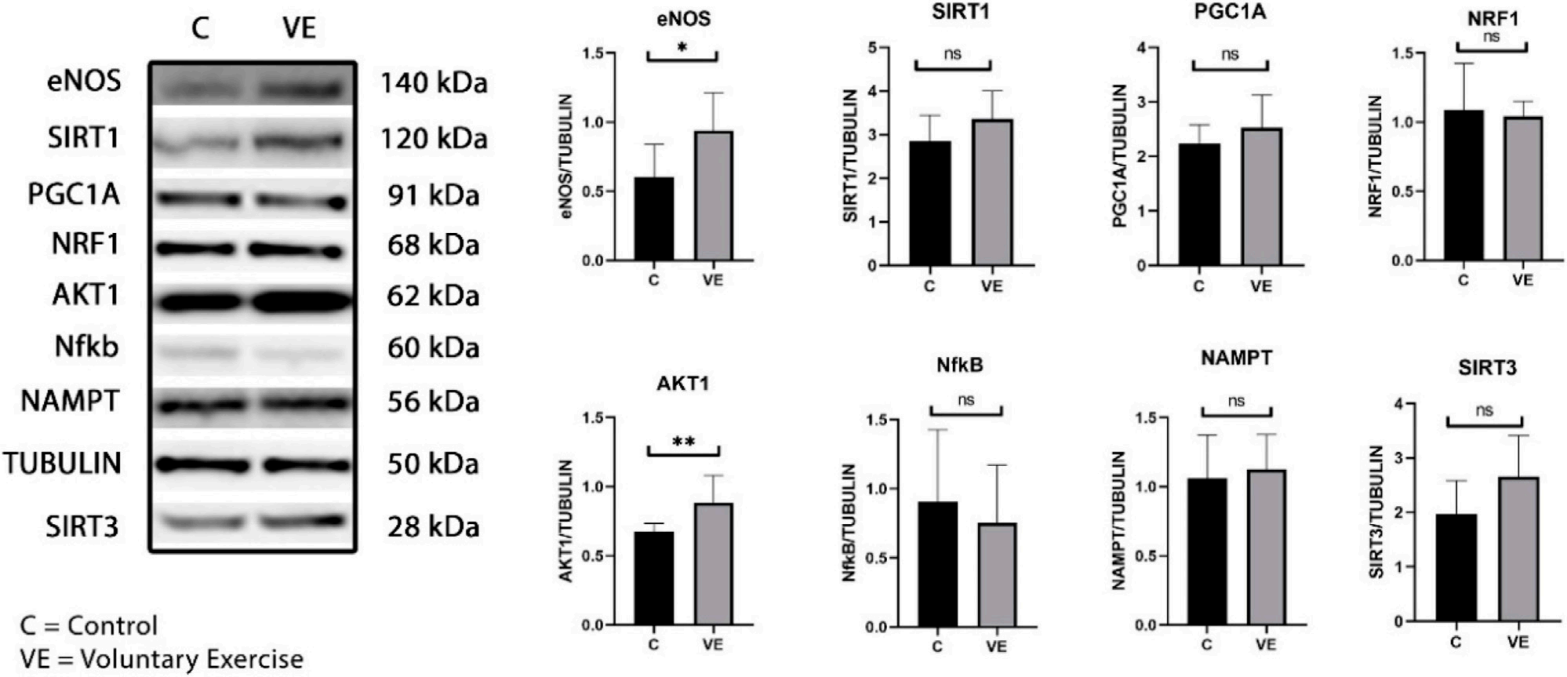
Immunoblot analysis of selected proteins of small intestine of control and exercised animals.

The Western blot analysis of the intestine samples revealed increased levels of eNOS and Akt, while no significant changes were observed at the protein levels of SIRT1, SIRT3, NRF1, PGC-1a, NF-kB, and NAMPT (Figure 3).

Immunoblot signals show that voluntary exercise increased eNOS and Akt1 levels in the small intestine. The results were normalized to tubulin and are shown as mean ± SD of the control (C, N = 6) and VE (N = 8) rats. The statistical significance was assessed using the two-way ANOVA.

**FIGURE 4:**
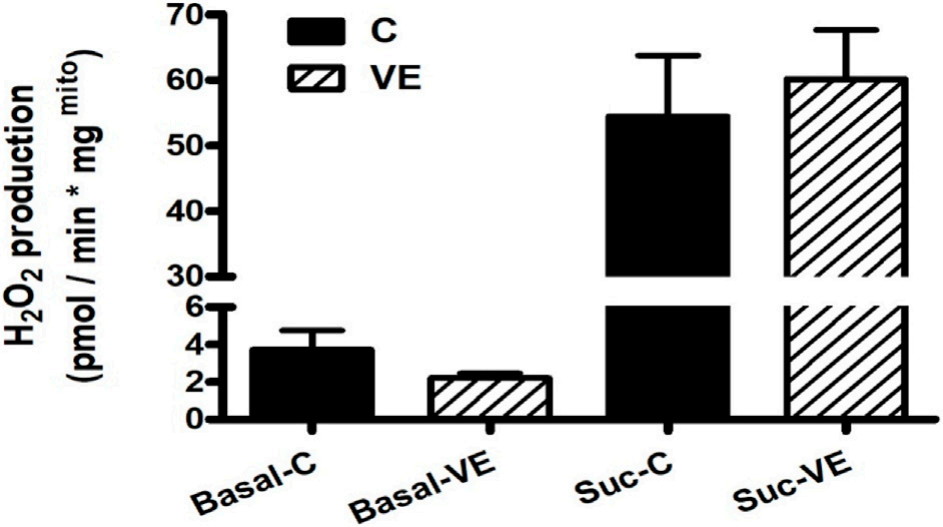
Effects of VE on mitochondrial ROS production in rat colon.

When ROS production was measured from freshly isolated mitochondria from the intestine, no significant difference was found between the C and VE groups (Figure 4).

Voluntary exercise resulted in no significant change in ROS production either before or after succinate administration. Rotenone treatment following succinate administration resulted in complete inhibition of ROS production in all cases. The results are mean ± SEM (N = 4–5). The statistical significance was assessed using an unpaired Student’s t-test.

**FIGURE 5:**
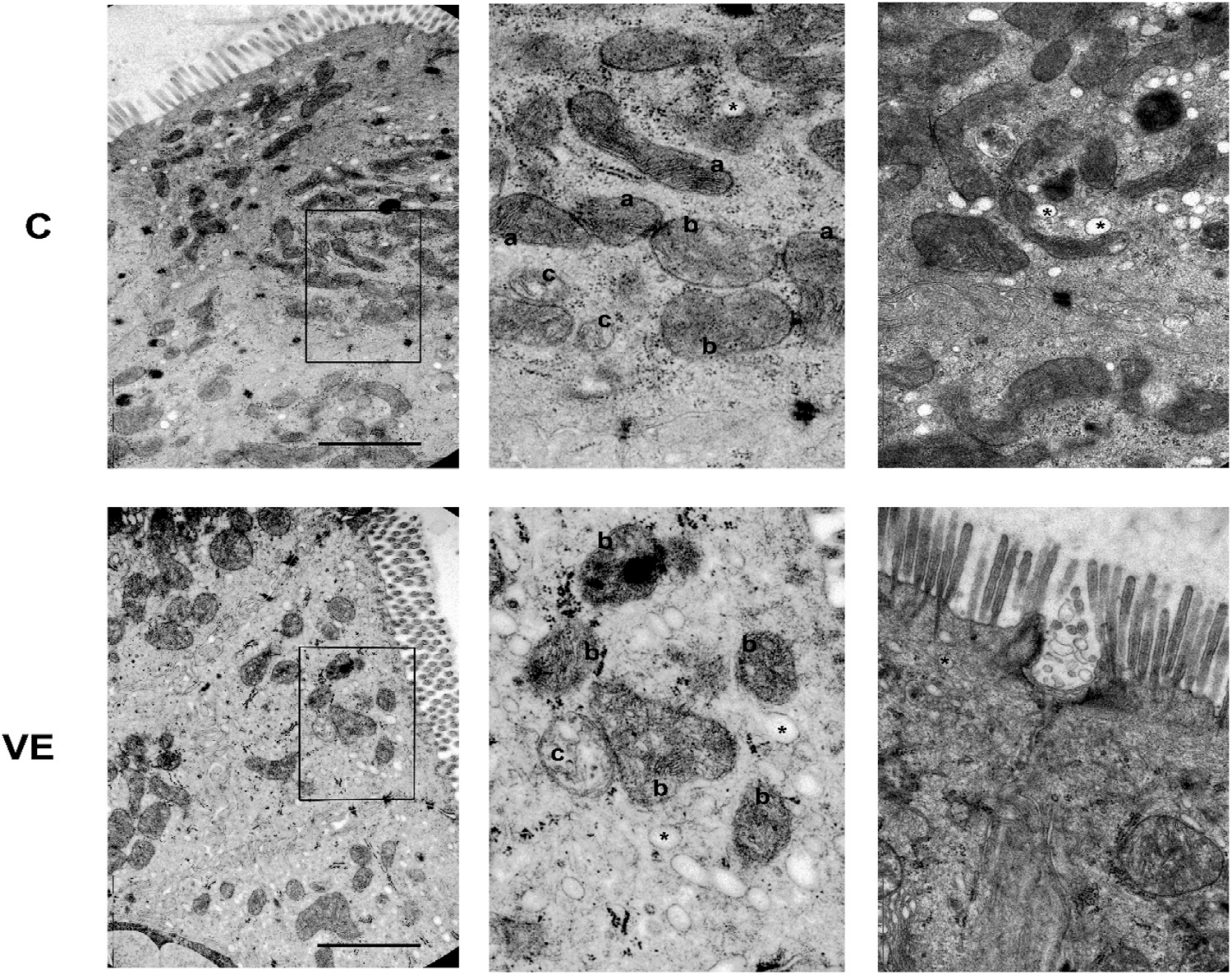
Representative electron micrographs of mitochondrial and vesicular alterations in the smooth muscle of colon caused by voluntary exercise. A Deep learning by APEER differentiated among the following groups that were defined based on colon mitochondrial repertoire: (a) normal, dense matrix with parallel narrow cristae; (b) disordered, still dense matrix but inordinate cristae with lipid droplets in the matrix; (c) swollen, extensive or complete loss of cristae and matrix density, significant loss of internal structure. (*) vesicula was also identified by APEER.

**FIGURE 6:**
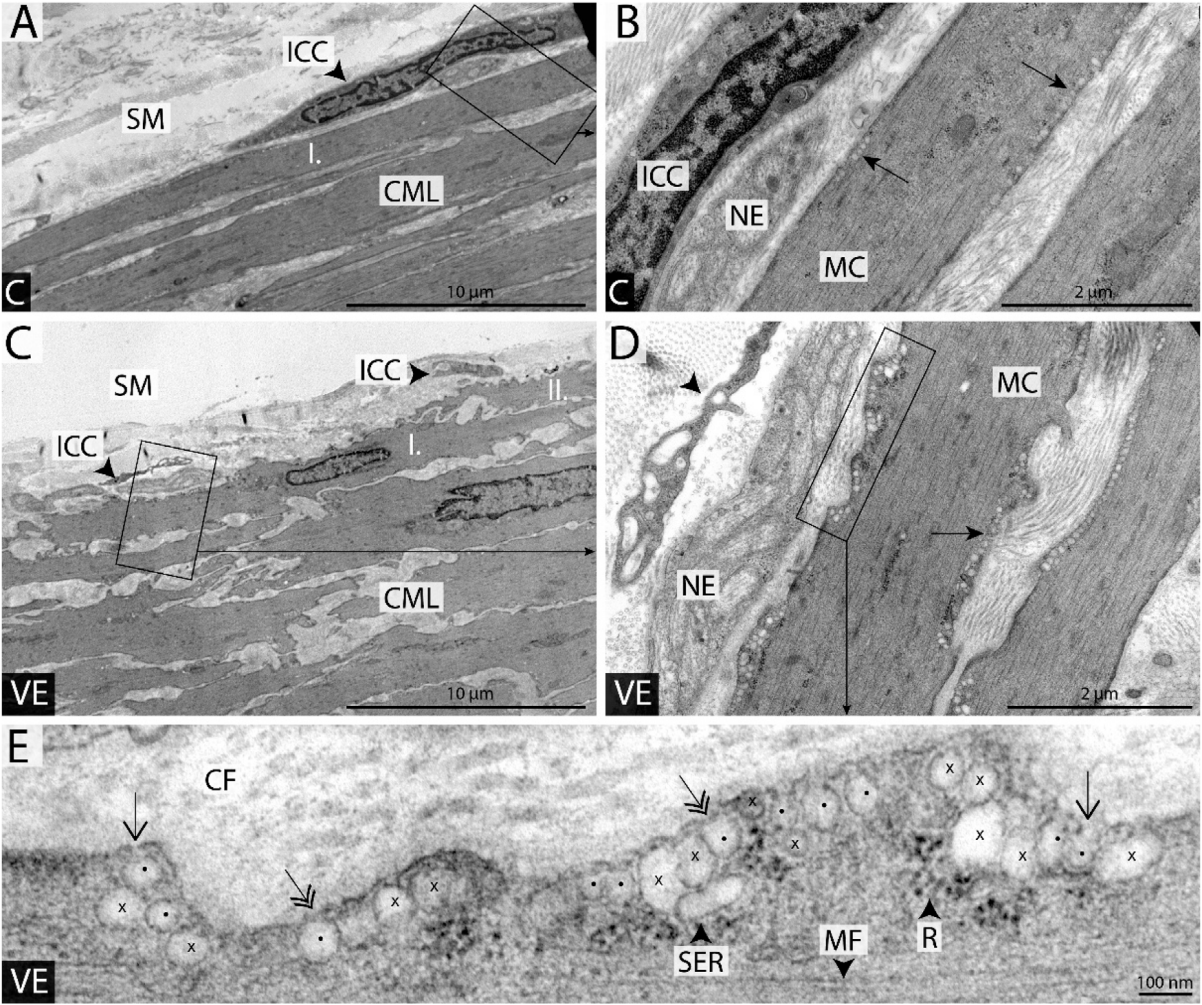
Representative colonic smooth muscle cells from sedentary and voluntary trained groups **(A, C)**. Localization and shape of the longitudinally sectioned innermost smooth muscle cells **(**I in **A)** and **(**I and II in **C)** on the submucosal surface of the circular muscle layer (CML). Whether the innermost cells form the submucosal surface, their one end always bends under the neighboring muscle cell; see overlapping cells marked by I and II in **(C)**. Interstitial cells of Cajal (ICC) form a network at the bottom of the submucosal layer (SM). **(B, D)** Groups of caveolae are clearly visible in the innermost smooth muscle cells (SMCs) just beneath the plasmalemma not only in the vicinity of nerve endings (EN) but on the opposite side as well (see arrows). **(E)** Demonstration of the method applied during the quantification of caveolae density; rows of caveolae (spots and crosses), smooth endoplasmic reticulum cistern (SER), ribosomes (R), and myofibrils (MF) in the peripheral sarcoplasm of muscle cell. Only flask-shaped membrane invaginations with visible openings to the cell surface (arrows) or with diaphragm covering the stoma (double-headed arrows) were considered in the quantification (see caveolae marked by spots). Membrane formations with elongated shape or missing connections to plasmalemma in the plane of the section were neglected (see crosses; CF, collagen fibers; VE, voluntary trained group; SED, sedentary group; arrowhead in **(D)**, the process of a Cajal cell; **(B, D, E)** are the magnified area of **(A, C, D)**, respectively; scale bars: 100 μm on **(A, C)**, 2 μm on **(B, D)**, 100 nm on **(E)**.

When the morphology of the mitochondria was studied using the electron microscopy data, it revealed that voluntary exercise increased the size of mitochondria, but at the same time, it also caused the elongation of normal mitochondria (Figures 5, 6).

The population of caveolae was evaluated using a microscope, and it appears that voluntary exercise increased the number of caveolae in the smooth muscle of the intestine (Figure 6).

**FIGURE 7:**
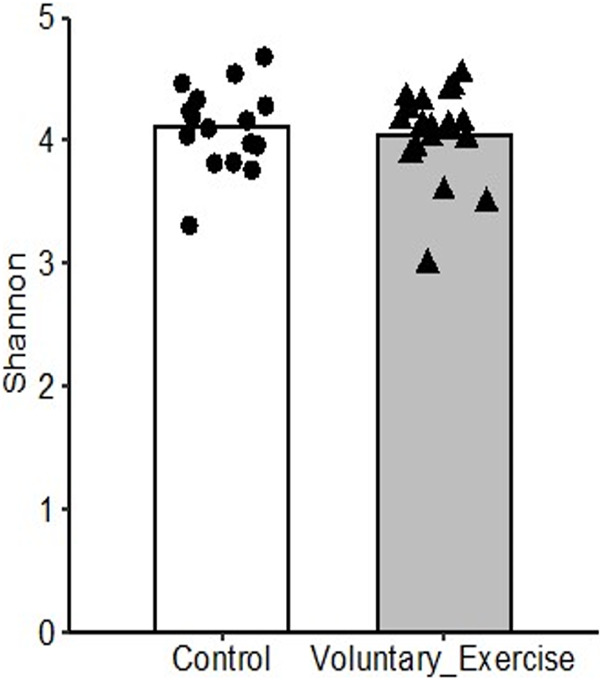
Shannon diversity index of the microbiome of trained and control rats.

The microbiome analysis showed a very similar Shannon diversity index of the bacterial community (Figure 7).

The Shannon diversity index was evaluated, and the results are shown in Figure 7. The Shannon diversity index of the microbiome was independent of the training status.

**FIGURE 8:**
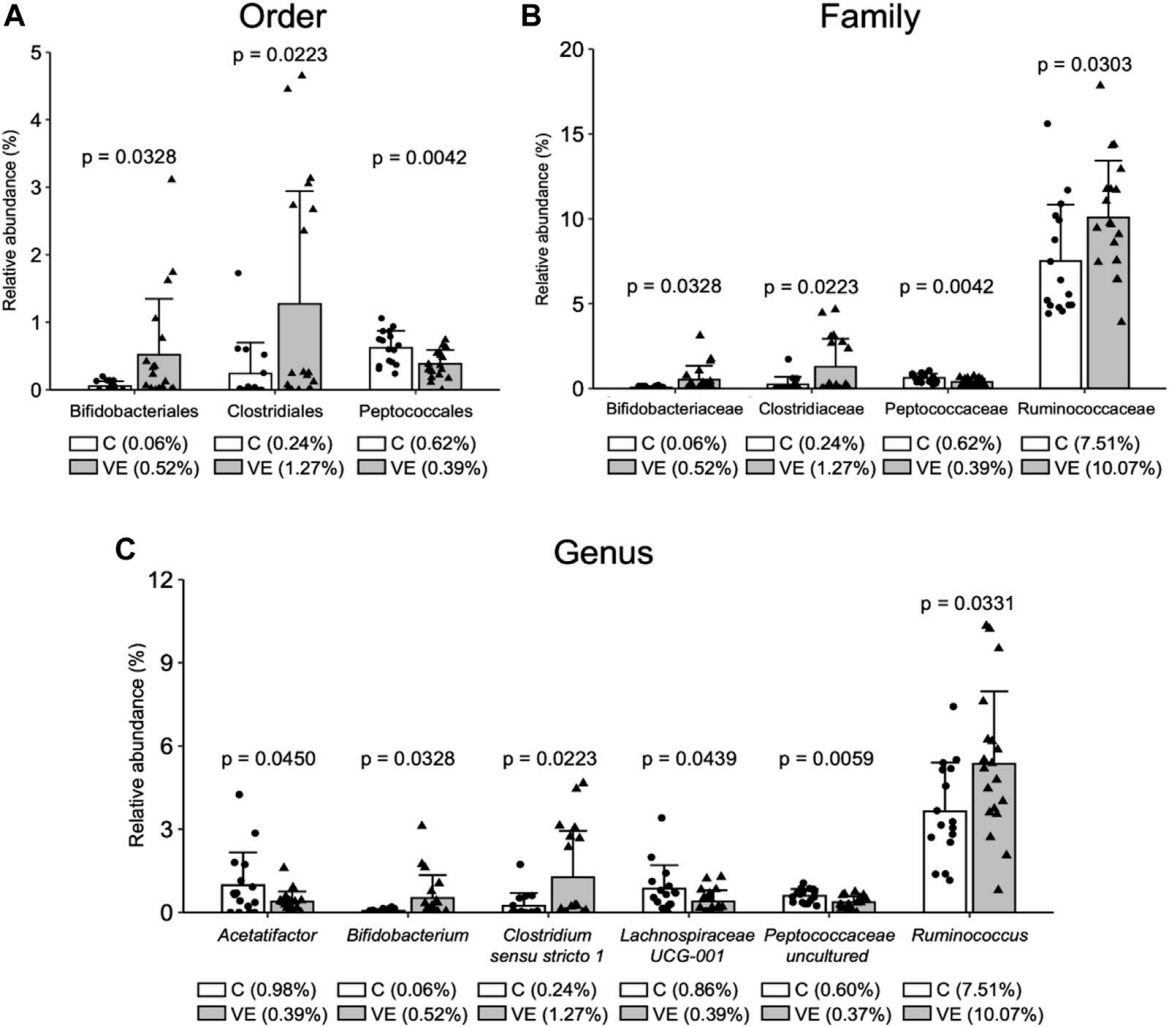
Relative abundance of bacterial community in gastrointestinal microbiome. Voluntary exercise changed the relative abundance of various bacterial communities in order **(A)**, family **(B)**, and genus **(C)** levels.

Voluntary exercise increased the relative abundance of Actinobacteria, which is one of the four major phyla of gut microbiota. At the family level, the relative abundance of *Bifidobacteria* and Ruminococcaceae increased (Figure 8), suggesting increased production of SCFA. Although NF-kB remained unchanged, the abundance of *B. Acetatifactor* decreased in the VE group, possibly compared to control animals.

The interrelationship of the measured parameters revealed that the lactate level negatively correlated with Akt and eNOS levels (r = −0.646 and r = −0.511, respectively) and positively correlated with NRF1 levels (r = 0.742). VO_2_max negatively correlated with spatial memory (r = −0.457). The abundance of Bifidobacteriales and Bifidobacteriaceae in the microbiome was associated with the level of VO_2_max (r = 0.436).

## Discussion

Regular exercise results in powerful systemic adaptation, which includes the brain–gut (intestine)–microbiome axis, and this is one of the first investigations that has studied the complexity of these interactions. It is well known that exercise can improve brain function (Radak et al., 2001; Sarga et al., 2013), and the involvement of the microbiome is also described (Mohle et al., 2016; Abraham et al., 2019). However, the possible involvement of mechanical and biochemical contribution of the intestine is not deeply understood. It has been shown that mild exercise accelerates, while severe exercise delays the emptying of the gastrointestinal tract (Read and Houghton, 1989). Here, we report that VE rats have higher levels of myoelectric activity of the gastrointestinal tract than control animals, but the difference did not reach levels of significance. In a human study, it was reported that exercise significantly accelerated gastric emptying but had no significant effect on small bowel transit time (Cammack et al., 1982). In the rat model, acute exercise delayed the gastric emptying of a liquid test meal by interfering with the acid–base balance (Silva et al., 2014). Moreover, it was observed that when rats were vagotomized, it resulted in gastric retention, suggesting a role of the vagal nerve in facilitating food movement and digestion in the stomach (Wang et al., 2010) and indicating a complex regulatory mechanism behind gastrointestinal motility.

During exercise bouts, the blood flow is downregulated in the intestine, while during rest, it is normalized, but this type of change in blood flow did not cause an increased generation of mitochondrial ROS generation. On the other hand, we observed an elevated protein level of eNOS, which was examined to assess whether a similar adaptation associated with the endothelium occurs in the intestine, as observed in the vasculature of skeletal and cardiac muscles during exercise training. Exercise sessions can lead to a decrease in pH, which subsequently reduces the contractile function in the intestine (Yartsev et al., 2002). It has been demonstrated that this acidosis can enhance the eNOS-derived NO signaling pathway in the vascular wall (Mohanty et al., 2018). Augmented eNOS signaling has the potential to stimulate the activation of VEGF and promote increases in microcirculation within the intestine (Abraldes et al., 2006), while also being associated with mitochondrial biogenesis (Vettor et al., 2014).

The gastrointestinal tract represents a very complex ecosystem, with various interactions of the digestive system with immune cells, endocrine cells, and nerve cells from a great distance. The intestine is in close proximity to the gastrointestinal microbiome; therefore, we examined the possible relationship between them. Here, we found that Akt protein content significantly increased in the intestine and correlated to the bifidobacterial abundance of the fecal microbiome. Akt is involved in peripheral glucose uptake and insulin sensitivity; moreover, Akt signaling in the brain is associated with depression (Pomytkin et al., 2015). It has been suggested that *Bifidobacterium*, which is a genus of anaerobic bacteria, and some of the strains used as probiotics have a potent anti-inflammatory role (Delzenne and Cani, 2008), preventing local intestinal inflammation by lowering the levels of lipopolysaccharide which can readily cause the formation of a leaky gut (Bhatia et al., 2022). The beneficial effects of exercise on insulin sensitivity are well known, therefore at least a part can be mediated by the Akt–*Bifidobacteria* axis, which could even have an antidepressant role (Pomytkin et al., 2015).

One of the other interesting findings of the present study is the adaptive response of SMCs to exercise with the increased formation of caveolae. Caveolae are generally abundant in the smooth muscle and characterized as flask-shaped invaginations connected to the plasmalemma by a neck-like structure and separated from the general extracellular space by the basement membrane involved in force transmission signal transduction pathways, membrane organization, and protein trafficking (Gabella, 1984; Hnasko and Lisanti, 2003). Here, we report for the first time to our knowledge that exercise increases the number of caveolae in the SMCs of the intestine, which could suggest enhanced force generation and signaling activity, thereby showing the beneficial effects of voluntary exercise on gastrointestinal health.

In studying the systemic effects of exercise-associated adaptation and the possible interrelationship between organs, the results of the correlation matrix revealed that animals whose VO_2_max level was higher after the 6 weeks of voluntary exercise period could locate the hidden island in the Morris maze test in a shorter period of time than did the rats with lower VO_2_max levels. This is in accordance with earlier observations (Marton et al., 2016; Liu et al., 2022). The other interesting correlation is between the level of lactic acid that was measured after VO_2_max texts and the NRF1 levels in the intestine. It was reported that the running time of marathon runners correlated with the abundance of *Veillonella atypica* from their stool samples (Scheiman et al., 2019). The authors suggested that athletes’ running performances increased because gut microbiota took up lactate and disposed of it as propionate. Interestingly, Brooks (2020), who proposed the lactate shuttle theory, suggest that the authors of previous study had a wrong conclusion, and he proposed that gut supplied lactate a fermentation product that is exported via sodium-mediated monocarboxylate transporters thus supporting athletes’ efforts. Either way is correct; the interrelationship between lactic acid and NRF1 could mean that the changes in the lactate/pyruvate ratio, which directly affects NAD/NADH ratio and the redox state, upregulate NRF1.

The abundance of Bifidobacteriales and Bifidobacteriaceae in the microbiome is associated with the level of VO_2_max, adding new bacterial strains to performance-linked bacteria community (Soares et al., 2019; Huang et al., 2020). However, it must be mentioned that when comparing the fecal microbiota composition and the levels of short-chain fatty acids and lactate in three of the most widely used animal models (mice, rats and non-human primates) with human samples, significant similarities and differences were observed (Nagpal et al., 2018). Therefore, it is crucial to exercise great caution when extrapolating animal results to human.

In conclusion, the present study investigated the brain–exercise–intestine–microbiome axis, and although we could not find exercise-induced motility, exercise increased Akt levels in the intestine, which was associated with the greater abundance of *Bifidobacteria* in the microbiome. It cannot be excluded that this adaptive response could be responsible for the increased insulin sensitivity of trained animals, which requires further investigation, but our results emphasize the importance of the interaction of the intestinal wall and gastrointestinal microbiome of exercise-induced systemic adaptation.

## Data Availability

The data sets presented in this study can be found in online repositories. The names of the repository/repositories and accession number(s) can be found at https://www.ebi.ac.uk/ena/browser/text-search?query=%20PRJEB62118.
