# Supplementary figures and images for: Voluntary exercise does not increase gastrointestinal motility but increases spatial memory, intestinal eNOS, Akt levels, and Bifidobacteria abundance in the microbiome
Source: Front Physiol. 2023 Aug 16;14:1173636. doi: 10.3389/fphys.2023.1173636 (PMC10468588; doi:10.3389/fphys.2023.1173636)

## Slide 1
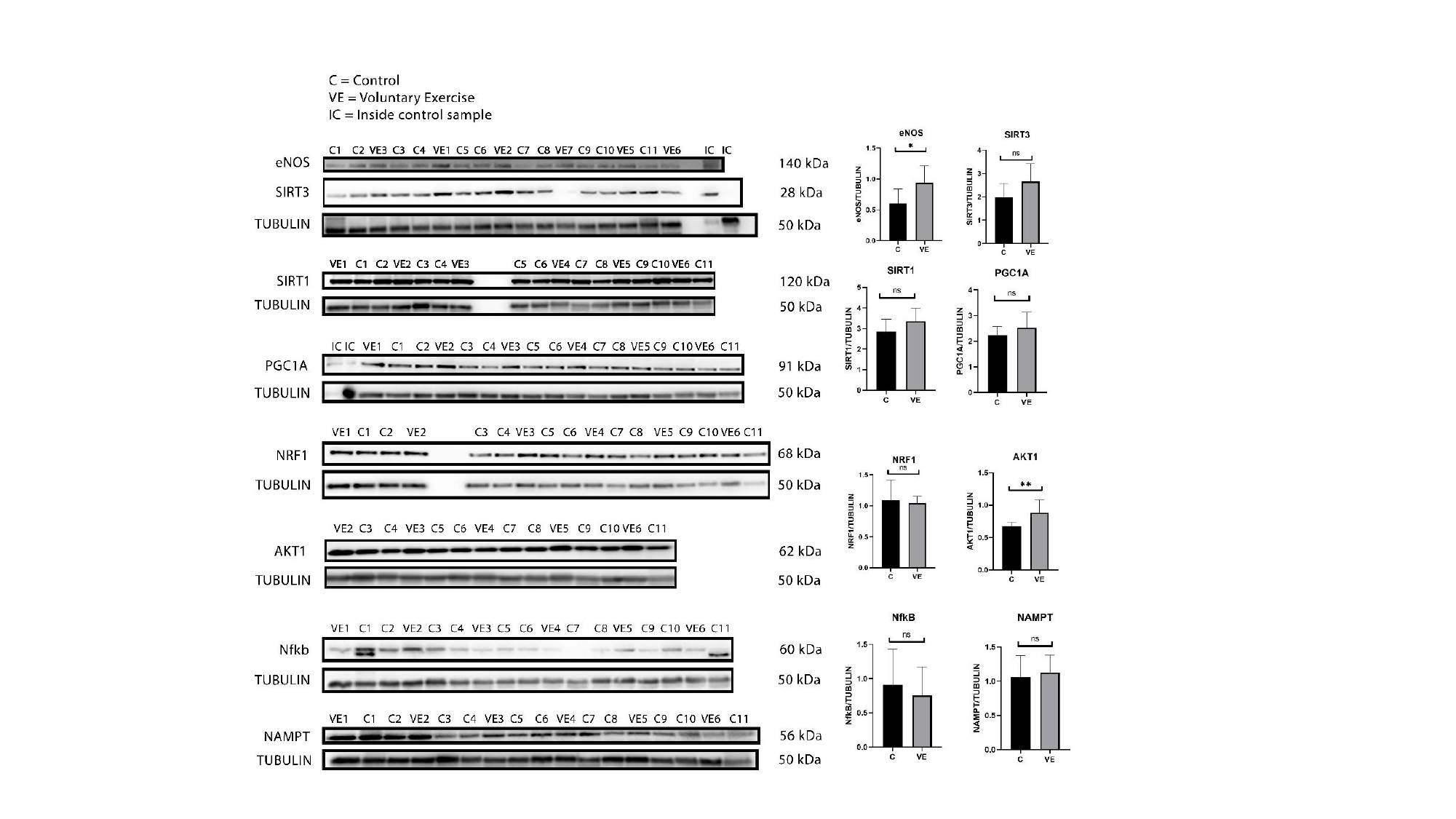

Supplement: Supplementary file 1 [file Presentation1.PPTX]
